# Regulation of Platelet-Derived ADAM17: A Biomarker Approach for Breast Cancer?

**DOI:** 10.3390/diagnostics11071188

**Published:** 2021-06-30

**Authors:** Yanjun Zhou, Jonas S. Heitmann, Korbinian N. Kropp, Martina Hinterleitner, André Koch, Andreas D. Hartkopf, Helmut R. Salih, Clemens Hinterleitner, Stefanie Maurer

**Affiliations:** 1 Cluster of Excellence iFIT (EXC 2180) “Image-Guided and Functionally Instructed Tumor Therapies”, University of Tuebingen, 72076 Tuebingen, Germany; Yanjun.Zhou@med.uni-tuebingen.de (Y.Z.); Jonas.Heitmann@med.uni-tuebingen.de (J.S.H.); Martina.Hinterleitner@med.uni-tuebingen.de (M.H.); Helmut.Salih@med.uni-tuebingen.de (H.R.S.); Stefanie.Maurer@med.uni-tuebingen.de (S.M.); 2 Clinical Collaboration Unit Translational Immunology, German Cancer Consortium (DKTK), Department of Internal Medicine, University Hospital Tuebingen, 72076 Tuebingen, Germany; 3 Department of Hematology, Medical Oncology and Pneumology, University Medical Center of Mainz, 55131 Mainz, Germany; korkropp@uni-mainz.de; 4 Department of Medical Oncology and Pneumology (Internal Medicine VIII), University Hospital Tuebingen, 72076 Tuebingen, Germany; 5 Department of Obstetrics and Gynecology, University Hospital Tuebingen, 72076 Tuebingen, Germany; Andre.Koch@med.uni-tuebingen.de (A.K.); Andreas.hartkopf@med.uni-tuebingen.de (A.D.H.); 6 Department of Radiology, Memorial Sloan Kettering Cancer Center, New York, NY 10065, USA

**Keywords:** platelets, breast cancer, biomarker, ADAM17, metastasis

## Abstract

Tumor progression and metastasis are critically dependent on the tumor microenvironment. A disintegrin and metalloproteinase 17 (ADAM17) is associated with shedding of several substrates involved in tumor progression and known to be expressed by platelets of healthy donors and patients with solid tumors. Here, we report that platelet-derived ADAM17 (pADAM17) is regulated upon platelet activation of breast cancer patients, but not of healthy individuals. The observed downregulation of pADAM17 on platelets of cancer patients correlated with clinical parameters related to tumor progression including tumor stage and the occurrence of metastasis. Our data identify an association between platelet activation, modulation of platelet-derived ADAM17, and metastasis. In conclusion, we demonstrate that further development of pADAM17 as a liquid biomarker is warranted for monitoring disease progression in breast cancer.

## 1. Introduction

With the development of early screening programs and improvement of comprehensive therapy regimen including surgery, chemotherapy, endocrine therapy, and monoclonal antibody therapy, the survival rate of breast cancer patients has dramatically increased [1]. Yet, almost 30% of patients who have initially been diagnosed with early-stage breast cancer will develop metastatic and life-threatening disease. The process of tumor metastasis includes local invasion of the primary tumor cells through surrounding extracellular matrix and stromal cell layers, intravasation into the lumina of blood or lymphatic vessels, survival in circulation, arrest at distant organ sites, extravasation into the parenchyma of distant tissues, survival in new environments, and metastatic colonization [2]. Platelets can participate in and facilitate all these processes of tumor cell dissemination [3]. A disintegrin and metalloproteinases (ADAMs), particularly ADAM17 (also named tumor necrosis factor (TNF) alpha converting enzyme, TACE), are localized intracellularly and are expressed at a low abundance on the cell surface of various cell types [4,5,6]. ADAM17 is a protein extracellular-region (ectodomain) sheddase responsible for cleavage of substrates that are, among others, substantially involved in cancer progression, including Jagged 1 promoting cancer stem cell phenotypes [7,8], neuregulin 1 promoting metastasis [9,10], and vascular endothelial growth factor receptor inducing tumor angiogenesis [11,12,13]. Recent evidence has revealed that enhanced ADAM17 expression on the tumor cell surface is associated with tumorigenesis, invasiveness, metastasis, and drug resistance in various cancers [14,15]. Our previous work demonstrated that ADAM17 is present on platelets (pADAM17) and contributes to immune evasion of metastasizing tumor cells [16]. However, the regulation of pADAM17 in tumor patients and its association with tumor progression have not yet been investigated. In this study, we evaluated ADAM17 expression in platelets and found a specific downmodulation on activated platelets of breast cancer patients. We report that a high extent of ADAM17 downregulation (∆pADAM17^high^) during platelet activation in patients—which may occur, among others, after their interaction with metastasizing tumor cells—correlates with certain tumor stages and the occurrence of metastases in breast cancer patients. Our data suggest to utilize pADAM17 as novel biomarker that can be readily assessed in liquid biopsies.

## 2. Materials and Methods

### 2.1. Reagents

Paraformaldehyde (PFA) was from Affymetrix (Santa Clara, CA, USA). Anti-human ADAM17 antibody and the respective isotype control were purchased from RD Systems (Minneapolis, MN, USA). CD41a-PeCy5 and CD62P-FITC were from BD Pharmingen (San Diego, CA, USA). The goat anti-mouse PE conjugate was from Dako (Glostrup, Denmark).

### 2.2. Patients

During 2019–2020, blood samples from 70 breast cancer patients treated at the Department of Obstetrics and Gynecology and the Department of Medical Oncology and Pneumology were included in our prospective study. Blood was only taken once for each donor. Written informed consent in accordance with the Helsinki protocol was given in all cases. The patient characteristics in detail are given in Table 1. The study was approved by the IRB (ethics committee of the Faculty of Medicine of the Eberhard Karls Universität Tuebingen) of the University Hospital Tuebingen and was conducted in accordance with the Declaration of Helsinki; reference number 13/2007V.

### 2.3. Preparation of Platelets

Citrated blood from breast cancer patients and healthy donors who did not take any anticoagulants for at least 10 days before blood collection was centrifuged (20 min, 120× *g* without brake) and the upper layer was harvested as platelet-rich plasma [17]. Subsequently, the fixation of platelets was performed by incubation of platelet-rich plasma in PFA (final concentration for PFA 2%) for 10 min at room temperature followed by two washing steps with phosphate buffer saline containing 1% fetal calf serum (1200× *g*, 10 min) [18,19]. The fixed platelets were stored at 4 °C for a maximum of one week before further analyses.

### 2.4. Flow Cytometry

For flow cytometric analyses, an unlabeled anti-ADAM17 antibody followed by a goat anti-mouse PE (1:100) as a secondary antibody or fluorophore-conjugated antibodies were used at saturating concentrations. The analysis was performed using a FACS Fortessa (BD Biosciences, Heidelberg, Germany). For reasons of uniformity and as resting and activated platelets display different levels of autofluorescence, the expression levels of target molecules are indicated as percent positive events rather than mean/specific fluorescence intensities (MFI/SFI). Percent positive platelets were calculated as follows: “percent surface expression obtained with specific antibody”—“percent surface expression obtained with isotype control”. Platelets were selected by CD41a+ and CD62P- (resting) or CD62P+ (activated). ∆pADAM17 was defined as follows: “percent ADAM17 expression in resting platelet”—“percent ADAM17 expression in activated platelet. The 75th percentile is regarded as ∆pADAM17 high, while all other valued are defined as ∆pADAM17 low.

### 2.5. Statistics

For the continuous variables, Student’s *t*-test and Mann–Whitney U test were used. CD62P expression level and pADAM17 and ∆pADAM17 expression were analyzed using simple linear regression analysis. All statistical tests were considered significant when *p* was below 0.05.

## 3. Results

### 3.1. Regulation of pADAM17 Expression during Platelet Activation

**Figure 1:**
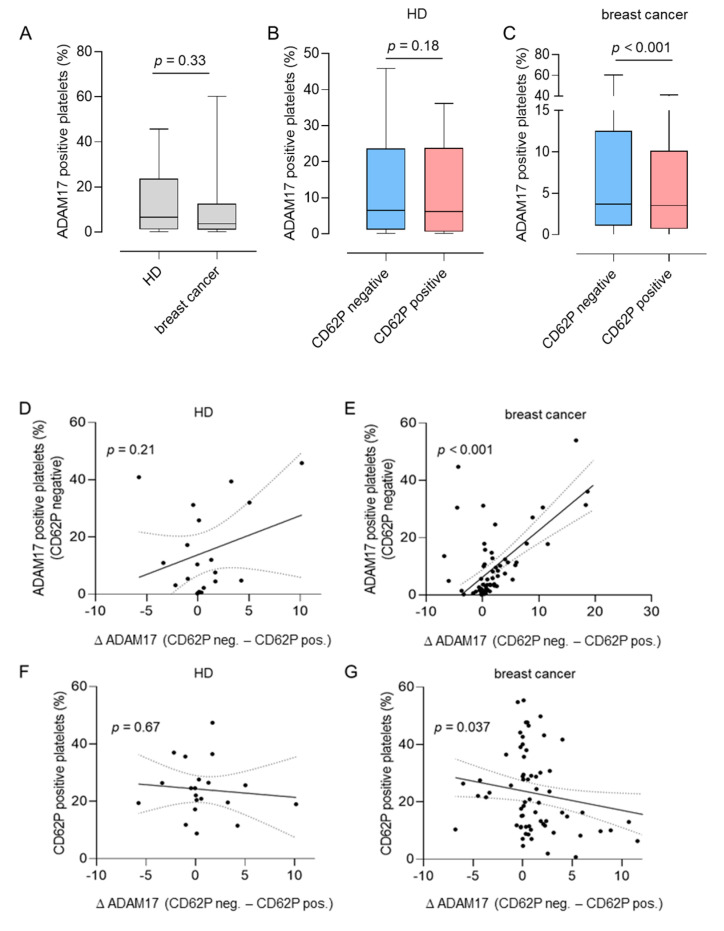
Platelet-derived A disintegrin and metalloproteinase 17 (pADAM17) upon platelet activation. (**A**) ADAM17 surface levels in resting (CD62P negative) platelets from 20 healthy donors (HDs) and 79 breast cancer patients. (**B**,**C**) ADAM17 surface levels in resting (CD62P negative) and activated (CD62P positive) platelets ex vivo from HD (**B**) or breast cancer patients (**C**). The expression levels of ADAM17 on resting or activated platelets in a given donor are connected. (**D**,**E**) Regulation of pADAM17 (∆pADAM17, defined as “the percentage of ADAM17 positive, CD62P negative platelets—the percentage of ADAM17 positive, CD62P positive platelets”) in HD (**D**) and breast cancer patients (**E)** with regards to the basal pADAM17 expression levels (percentage of pADAM17 positive and CD62P negative platelets). (**F**,**G**) The extent of pADAM17 down-regulation upon platelet activation (∆pADAM17) was analyzed in HD (**F**) and breast cancer patients (**G**) with regards to CD62P expression. Simple linear regression was used for statistical analysis in (**D**–**G**).

While ADAM17 has been described on platelets, little is known about the regulation and function of pADAM17 in the context of solid tumors [13]. In a first step, we determined the expression of ADAM17 on platelets of 20 healthy donors (HD) and 79 breast cancer patients using flow cytometry (Figure 1A–C). The clinical characteristics of 70 breast cancer patients included in this study are presented in Table 1.

Notably, HD and breast cancer patients displayed comparable total levels of pADAM17 (Figure 1A). In breast cancer patients, ADAM17 expression on the surface of activated platelets was lower compared with resting (CD62P-negative) platelets; this was not observed with HD (*p* < 0.001, Figure 1B,C).

Pre-existing platelet activation levels were similar in both HD and breast cancer patients. Basal pADAM17 expression of resting platelets from patients was significantly associated with the extent of pADAM17 downregulation (∆pADAM17: “percent ADAM17 expression in resting platelet”—“percent ADAM17 expression in activated platelet”) upon platelet activation in patients, which was again not observed with platelets derived from HD (*p* < 0.001, Figure 1D,E). Whereas ∆pADAM17 is not regulated in HD (Figure 1F), an inverse association of pADAM17 downmodulation was observed with the basal activation level in patient-derived platelets (*p* = 0.037, Figure 1G). In summary, ADAM17 expression appears to be regulated on the surface of platelets in the context of malignant disease.

### 3.2. Asscociation of pADAM17 with Platelet Activation and Clinical Parameters

**Figure 2:**
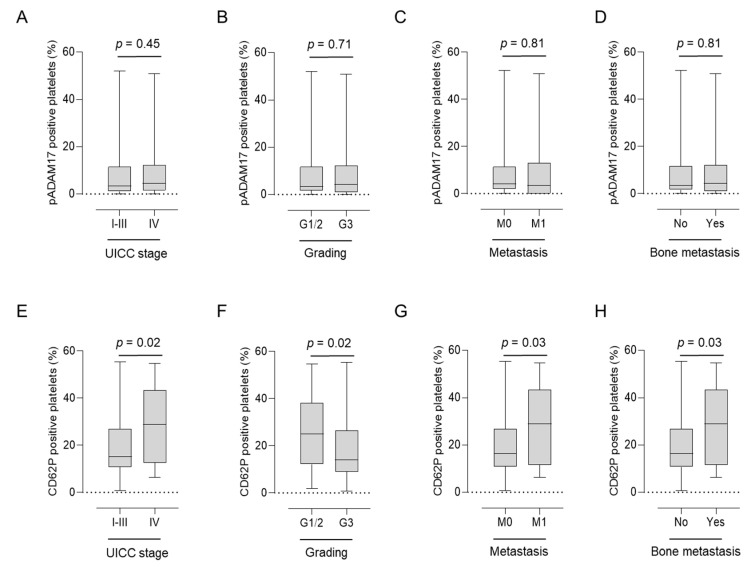
Association of pADAM17 (**A**–**D**) and platelet-CD62P (**E**–**H**) expression levels of 70 breast cancer patients with regards to Union for International Cancer Control (UICC) stage (**A**,**E**), G grade (**B**,**F**), M stage (**C**,**G**), and the occurrence of bone metastasis (**D**,**H**).

As pADAM17 levels were regulated in patients with solid tumors, we further assessed their association with prognostic clinical parameters. pADAM17 expression on platelets ex vivo did not correlate with Union for International Cancer Control (UICC) stage, tumor grading (G), or the occurrence of (bone) metastasis (Figure 2A–D). However, these parameters were clearly associated with platelet activation (CD62P expression). Whereas a higher activation status of patient-derived platelets was connected to more advanced UICC stage (*p* = 0.02), the occurrence of metastasis (*p* = 0.02), and bone metastasis (*p* = 0.03), higher tumor grade (G) was negatively associated with platelet activation (*p* = 0.02) (Figure 2E–H).

In contrast, no significant correlation was observed between pADAM17 levels and histological type, molecular subtype, T stage, or N stage (Supplementary Figure S1). Notably, pADAM17 levels tended to be particularly low in ductal carcinoma in situ (DCIS) and mucinous breast cancer, whereas expression tended to be highest in invasive lobular carcinoma (ILC).

Moreover, pADAM17 expression level appeared not be associated with hormone receptor status and proliferation index in our cohort (Supplementary Figure S2). Although platelet activation was connected to UICC stage, G grade, and the occurrence of metastasis, it was not associated with T, N, G or UICC classifications; ER, PR, or HER2 receptor status; or Ki67 tumor proliferation index (Supplementary Figure S3).

### 3.3. Association of ∆pADAM17 with Metastasis in Breast Cancer Patients

**Figure 3:**
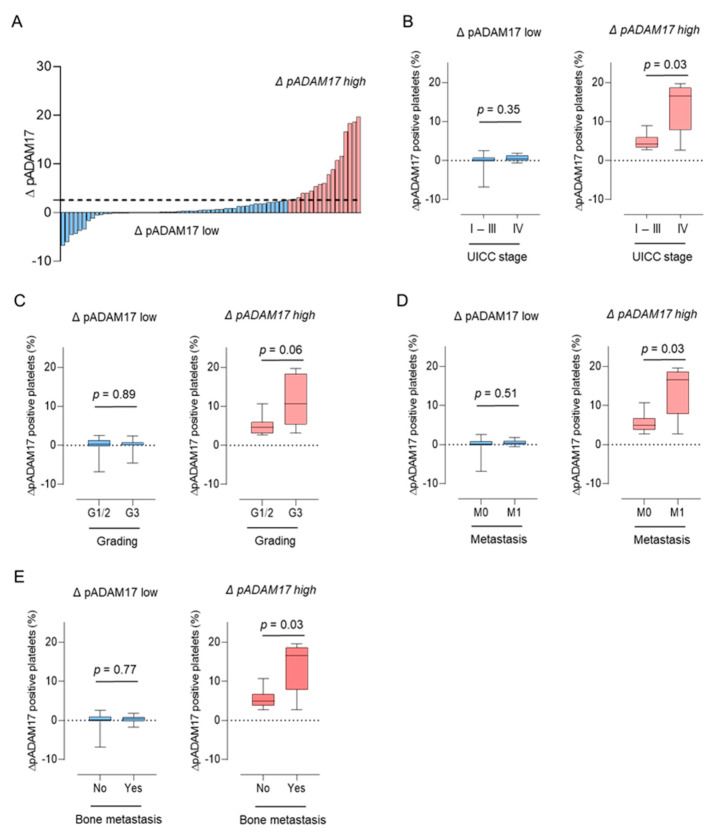
Correlation of pADAM17 downregulation (∆pADAM17) with clinical parameters in our breast cancer cohort. (**A**) Extent of ∆pADAM17 (defined as “the percentage of ADAM17 positive, CD62P negative platelets—the percentage of ADAM17 positive, CD62P positive platelets”). The 75th percentile is regarded as ∆pADAM17 high (red), all other values are defined as ∆pADAM17 low (blue). (**B**–**E**) Association of high ∆pADAM17 with Union for International Cancer Control (UICC) stage (**B**), G grade (**C**), the occurrence of metastasis (M stage) (**D**), and bone metastasis (**E**).

Our observation that pADAM17 does not correlate with tumor characteristics, but is regulated in breast cancer patients during platelet activation, leads us to further analyze the regulation of pADAM17 (∆pADAM17) in our breast cancer cohort in greater detail. In order to investigate a cohort in which pADAM17 was significantly modulated, we defined patient groups with ∆pADAM17^high^ and ∆pADAM17^low^ levels according to the respective quartile groups (Q), (Q1-3: ∆pADAM17^low^, Q4: ∆pADAM17^high^) (Figure 3A).

Whereas breast cancer patients without a relevant modulation of pADAM17 (∆pADAM17^low^) showed no difference in UICC stage (Figure 3B), tumor grading (Figure 3C), or the occurrence of metastasis (Figure 3D), patients displaying a strong modulation of pADAM17 (∆pADAM17^high^) had significantly higher UICC stages (*p* = 0.03, Figure 3B), higher tumor grading (*p* = 0.06, Figure 3C), higher rates of metastasis, and particularly bone metastasis (*p* = 0.03, Figure 3D,E). These findings indicate that ∆pADAM17 correlates with advanced disease stages and/or tumor progression and might serve as a potential biomarker for metastasis in breast cancer.

## 4. Discussion

Tumor progression and metastasis are highly complex processes that are modulated by a variety of factors, e.g., in the tumor microenvironment [3,20,21]. Among others, this comprises soluble factors like chemokines and cytokines, growth factors, adhesion molecules, and receptors that have also been described to be released as soluble forms by ectodomain shedding [22,23]. ADAM17, which is at the focus of this study, has been demonstrated as a key sheddase controlling ectodomain cleavage of various substrates involved in metastasis, tumor progression, and therapy resistance [9,24].

Here, we studied the expression of ADAM17 on the surface of platelets in the context of breast cancer. Our findings confirm and extend available data on the expression of ADAM17 on human platelets in general and in patients with solid tumors [16,25]. Although pADAM17 levels appear to be enhanced in lung carcinoma [16], no significant difference was obtained between healthy donors and breast cancer patients. A high inter-donor variability was observed, particularly in tumor patients, which might reflect certain “malignant platelet phenotypes” associated with distinct disease characteristics [26,27]. As expression and activity of various proteins on the platelet surface reportedly are influenced by platelet activation [28,29,30,31,32], we explored whether pADAM17 is also regulated by this process. In breast cancer patients, ADAM17 expression on the surface of activated platelets was lower compared with resting (CD62P-negative) platelets and pADAM17 downregulation was positively related to basal ADAM17 expression on resting platelets, which may indicate that pADAM17 expression is regulated upon platelet activation in the context of breast cancer. There are various possible mechanisms that may cause reduction of ADAM17 upon platelet activation, including secretion in extracellular vesicles (EVs) and/or proteolytic shedding. Scharfenberg and colleagues demonstrated that ADAM8 can shed both pro-ADAM17 and mature ADAM17 [33], and identified unique substrates of soluble ADAM17 that influence extracellular matrix degradation, e.g., fibronectin or N-cadherin [33]. Even if it was shown that ADAM17 can be proteolytically released from the cell surface, it is also possible that down-regulation of pADAM17 partially happens via release in the form of EVs. ADAM17-rich-EVs reportedly are active and may lead to pathologic conditions, such as vasculitis [6]. However, the functional relevance of pADAM17 in its membrane bound form, proteolytically cleaved or secreted in EVs, needs to be further investigated in the pathophysiology of solid tumors. In our study, the extent of pADAM17 downregulation is significantly related to pADAM17 expression in the resting platelet fraction. This was also observed as a tendency in healthy individuals. This led us to further study the role of both platelet activation (CD62P expression) and pADAM17 in breast cancer progression. Platelet P-selectin levels were found to be associated with higher UICC stages and the occurrence of (bone) metastasis, which is in line with previous studies reporting hyperactivity of platelets in metastatic tumor patients [34]. However, pADAM17 level were comparable according to the aforementioned parameters. Albeit, in cases where pADAM17 was highly regulated (∆pADAM17^high^), we observed a positive correlation of pADAM17 downregulation and tumor progression as revealed by UICC stage, G grade, the occurrence of metastasis, and bone metastasis. However, the data provided in our study cannot finally confirm ΔpADAM17 as a biomarker to monitor disease progression in breast cancer. To address this, a larger prospective biomarker study in breast cancer is needed. As breast cancer is a heterogeneous disease, our study cohort of only 70 breast cancer patients is unfortunately not able to adequately analyze the role of platelet-derived ADAM17 in different breast cancer subgroups (Supplementary Figure S1). As a result, further prospective studies of a larger patient cohort are urgently needed to investigate pADAM17 with regard to HR, HER2, and TNBC in more detail.

It is reported that ADAM17 can enhance breast cancer cells’ invasion and proliferation in vitro and promote breast cancer cell malignant phenotype [14,35,36]. Moreover, ADAM17 has been described to be involved in EGF-R- mediated IL-6 synthesis and tumorigenesis in colorectal cancer [37]. In hepatocellular carcinoma (HCC), ADAM17 seems to be involved in the development of HCC invasion and metastasis [38]. However, as the regulation and pathophysiology of ADAM17 in cancer and especially in tumor-educated platelets is complex, further elucidation is warranted.

Together, we demonstrate here that pADAM17 associates with platelet activation. More importantly, we identified an association of the extent of pADAM17 change (∆pADAM17) upon platelet activation with clinical parameters in breast cancer, indicating that downregulation of pADAM17 might be associated with tumor progression and metastasis. While several questions on the pathophysiologic role of pADAM17 and its regulation during malignant disease warrant further elucidation, our data provide a rationale to exploit ∆pADAM17 as a biomarker in breast cancer.

## Figures and Tables

**Table 1:** Patient characteristics.

Patient Characteristics	Total (*n* = 70)
Age	
Age in years, mean—yr. ± SD	60.3 ± 11.9
(range)	(27 to 87)
Gender	
Female, *n* (%)	69 (98.6)
TNM classification, *n* (%)	
Tumor size	
T0	2 (2.9)
T1	23 (32.9)
T2	31 (44.3)
T3	8 (11.4)
T4	5 (7.1)
unknown	1 (1.4)
Regional node	
N0	39 (55.7)
N1	18 (25.7)
N2	6 (8.6)
N3	2 (2.9)
unknown	5 (7.1)
Metastasis	
M0	45 (64.3)
M1	25 (35.7)
UICC stage, *n* (%)	
0	2 (2.9)
1	21 (30)
2	15 (21.4)
3	7 (10)
4	25 (35.7)
Localization of primary tumor, *n* (%)	
Right	25 (35.7)
Left	44 (62.9)
Bilateral	1 (1.4)
Histological grading, *n* (%)	
G1	5 (7.1)
G2	33 (47.1)
G3	31(44.3)
unknown	1 (1.4)
Receptor status, *n* (%)	
ER-positive	57(81.4)
PR-positive	45(64.3)
HER2 receptor	
Positive	13 (18.6)

UICC, Union for International Cancer Control; ER, estrogen receptor; PR, progesterone receptor; HER, human epidermal growth factor receptor.

## Data Availability

The data presented in this study are available on request from the corresponding author.

## Supplementary Materials

The following are available online at https://www.mdpi.com/article/10.3390/diagnostics11071188/s1: Figure S1: Association of pADAM17 expression with tumor stages of breast cancer patients; Figure S2: Association of pADAM17 expression with receptor status and proliferation index of breast cancer patients; Figure S3: Association of platelet activation and clinical parameters; Figure S4: Association of pADAM17 modulation (ΔpADAM17) with clinical parameters in breast cancer.

## Abbreviations

The following abbreviations are used in this manuscript:

ADAM	A disintegrin and metalloproteinase
DCIS	Ductal carcinoma in situ
ER	Estrogen receptor
EV	Extracellular vesicle
HD	Healthy donor
HER	Human epidermal growth factor receptor
ILC	Invasive lobular carcinoma
pADAM17	Platelet-derived ADAM17
PFA	Paraformaldehyde
PR	Progesterone receptor
TACE	TNF alpha converting enzyme
TNF	Tumor necrosis factor
UICC	Union for International Cancer Control
