# Supplementary material for: Regulation of Platelet-Derived ADAM17: A Biomarker Approach for Breast Cancer?
Source: Diagnostics (Basel). 2021 Jun 30;11(7):1188. doi: 10.3390/diagnostics11071188 (PMC8305148; doi:10.3390/diagnostics11071188)
Supplement: Supplementary file 1 [file diagnostics-11-01188-s001.zip › Supplementary Material.pdf]

Supplementary Materials

Regulation of Platelet-Derived ADAM17: A Biomarker Approach for Breast Cancer?

Yanjun Zhou, Jonas S. Heitmann, Korbinian N. Kropp, Martina Hinterleitner, André Koch, Andreas D. Hartkopf, Helmut R. Salih, Clemens Hinterleitner and Stefanie Maurer

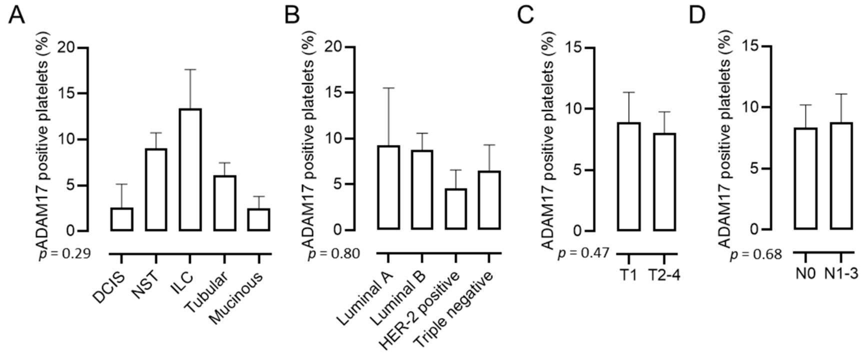

Figure S1. Association of pADAM17 expression with tumor stages of breast cancer patients. ADAM17 level with regard to histological type (**A**), molecular subtype (**B**), T stage (**C**), N stage (**D**), in 70 breast cancer patients are shown. Error bars represent standard error of the mean. DCIS, ductal carcinoma in situ; NST, no special type breast cancer; ILC, Invasive lobular carcinoma; Tubular, tubular breast cancer; Mucinous, mucinous breast cancer.

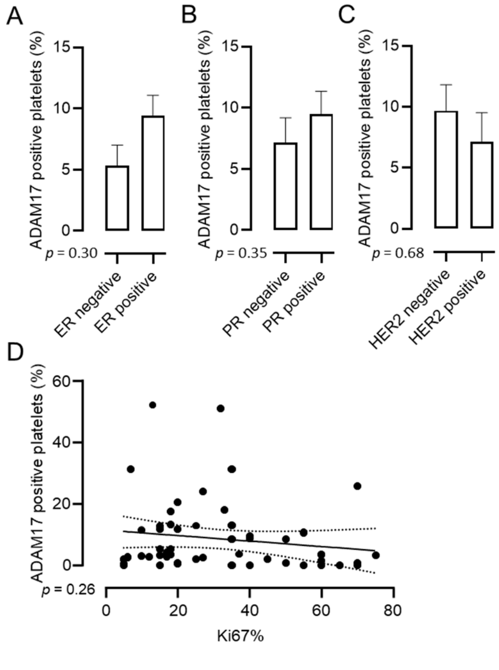

Figure S2. Association of pADAM17 expression with receptor status and proliferation index of 70 breast cancer patients. ADAM17 expression with regard to ER status (**A**), PR status (**B**), HER2 status (**C**), and Ki67% (**D**) in 70 breast cancer patients are shown. (**A-C**). Error bars represent standard error of the mean. Simple linear regression was used in (**D**). ER, estrogen receptor; PR, progesterone receptor; HER, human epidermal growth factor receptor.

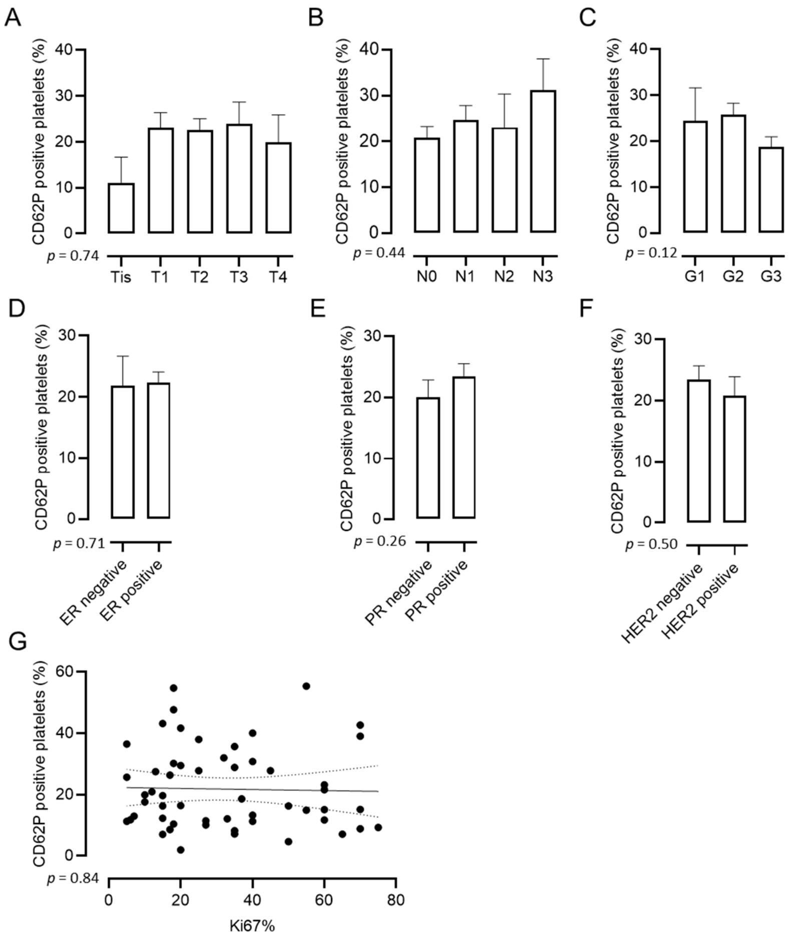

Figure S3. Association of platelet activation and clinical parameters. CD62P expression with regard to T stage (**A**), N stage (**B**), G grade (**C**), ER status (**D**), PR status (**E**), HER2 status (**F**), and Ki67% (**G**) in 70 breast cancer patients are shown. (**A-G**) Error bars represent standard error of the mean. Tis refers to carcinoma in situ.
