# Supplementary material for: Regulation of Platelet-Derived ADAM17: A Biomarker Approach for Breast Cancer?
Source: Diagnostics (Basel). 2021 Jun 30;11(7):1188. doi: 10.3390/diagnostics11071188 (PMC8305148; doi:10.3390/diagnostics11071188)
Supplement: Supplementary file 1 [file diagnostics-11-01188-s001.zip › Supplementary Table S1.pdf]

Supplementary Table S1. Patient characteristics.

Patient characteristics	Total (n=70)
Age	
Age in years, mean–yr. ± SD	60.3 ± 11.9
(range)	(27 to 87)
Gender	
Female, n (%)	69 (98.6)
TNM classification, n (%)	
Tumor size	
T0	2 (2.9)
T1	23 (32.9)
T2	31 (44.3)
T3	8 (11.4)
T4	5 (7.1)
unknown	1 (1.4)
Regional Node	
N0	39 (55.7)
N1	18 (25.7)
N2	6 (8.6)
N3	2 (2.9)
unknown	5 (7.1)
Metastasis	
M0	45 (64.3)
M1	25 (35.7)
UICC stage, n (%)	
0	2 (2.9)
1	21 (30)
2	15 (21.4)
3	7 (10)
4	25 (35.7)
Localization of primary tumor, n (%)	
Right	25 (35.7)
Left	44 (62.9)
Bilateral	1 (1.4)
Histological grading, n (%)	
G1	5 (7.1)
G2	33 (47.1)
G3	31(44.3)
unknown	1 (1.4)
Receptor status, n (%)	
ER-positive	57(81.4)
PR-positive	45(64.3)
HER2 receptor	
Positive	13 (18.6)

UICC, Union for International Cancer Control; ER, estrogen receptor; PR, progesterone receptor; HER, human epidermal growth factor receptor
